# Supplementary material for: Persistence with daily growth hormone among children and adolescents with growth hormone deficiency in Japan
Source: PLoS One. 2025 Aug 14;20(8):e0324728. doi: 10.1371/journal.pone.0324728 (PMC12352635; doi:10.1371/journal.pone.0324728)
Supplement: Attachment — Submitted filename: PLOSOne Submission_Response to Reviewers_Round 3_26Aug2024.docx [file pone.0324728.s001.docx]

**Dear Editors and Reviewers,**

**We would like to thank you for the time and consideration of our manuscript. We have taken your comments and have updated the manuscript accordingly.**

**The table below highlights the decisions we have made based on your comments.**

**Best,**

**Jane Loftus and fellow authors**

| Review Comment | Author responses |
| --- | --- |
| Reviewer #2 Comments | |
| As the authors described in the limitation section, the discontinuation thresholds may lead to an underestimation of non-persistence to therapy. I believe the setting of the cut-off value is a critical point for this study. In the critical practice, a gap of over 90 days is often occurred in patients treated with somatropin without discontinuation for the therapy. The authors should indicates more convincing arguments for the thresholds.  I am afraid that the authors did not address the reviewers’ comments. In the clinical practice, especially in Japan, a gap of 90 days between somatropin prescription fill dates is often occurred in GHD patients without discontinuation for the GH therapy. The discontinuation thresholds (>60 and >90 days) may lead to an underestimation of non-persistence to therapy. | **We would like to thank you for this comment. We have adjusted to the manuscript to indicate that the thresholds we have selected for this analysis are based on previous thresholds used in other somatropin persistence studies. Furthermore, we have added a 120-day gap definition, as suggested, for this analysis and have presented the results in Figure 4 and Table 2. As suspected, using the 120-day gap definition, fewer patients discontinue therapy; nonetheless, data indicate that between 16 and 28% of patients still discontinue treatment at 48 months even with this more conservative definition. We hope that presenting and describing the data in this manner using 3 thresholds provides useful information and insights with respect to prescribing patterns in Japan in this cohort of patients.**  **We have noted and cited that other studies on growth hormone therapy injections have used a 6-month gap definition for persistence. In this scenario, a 6-month definition would likely lead to our results overestimating the discontinuation and underestimating persistence, which we have noted in the limitations.** |
| Reviewer # 5 Comments | |
| The manuscript was revised twice before my review and I found it almost suitable for publication despite several limitations. One comment: There is a insulin in the lists of concomitant medication. However, in Japan, diabetes mellitus has been contraindicated in the GH treatment for GH deficiency till 2022. | **Thank you for this insight. Insulin was observed as one of the top 5 concomitant medications in the MDV database. We are unsure why this is the case, other than this may be an instance where clinical practice does not adhere to guideline therapies. Furthermore, as MDV is a hospital based dataset, there may be reasons outside of GHD that patients are receiving these treatments. Given these unknowns, the data was checked by the authors and no further actions were taken in revising the manuscript.** |
